# Biomembrane Structure and Material Properties Studied With Neutron Scattering

**DOI:** 10.3389/fchem.2021.642851

**Published:** 2021-04-27

**Authors:** Jacob J. Kinnun, Haden L. Scott, Rana Ashkar, John Katsaras

**Affiliations:** ^1^Large Scale Structures Group, Neutron Scattering Division, Oak Ridge National Laboratory, Oak Ridge, TN, United States; ^2^Oak Ridge National Laboratory, Shull-Wollan Center, Oak Ridge, TN, United States; ^3^Department of Physics, Virginia Tech, Blacksburg, VA, United States; ^4^Center for Soft Matter and Biological Physics, Virginia Tech, Blacksburg, VA, United States; ^5^Sample Environment Group, Neutron Scattering Division, Oak Ridge National Laboratory, Oak Ridge, TN, United States; ^6^Department of Physics and Astronomy, University of Tennessee, Knoxville, TN, United States

**Keywords:** neutron scattering, membrane biophysical properties, lipid domains/rafts, membrane dynamics, MD simulation

## Abstract

Cell membranes and their associated structures are dynamical supramolecular structures where different physiological processes take place. Detailed knowledge of their static and dynamic structures is therefore needed, to better understand membrane biology. The structure–function relationship is a basic tenet in biology and has been pursued using a range of different experimental approaches. In this review, we will discuss one approach, namely the use of neutron scattering techniques as applied, primarily, to model membrane systems composed of lipid bilayers. An advantage of neutron scattering, compared to other scattering techniques, is the differential sensitivity of neutrons to isotopes of hydrogen and, as a result, the relative ease of altering sample contrast by substituting protium for deuterium. This property makes neutrons an ideal probe for the study of hydrogen-rich materials, such as biomembranes. In this review article, we describe isotopic labeling studies of model and viable membranes, and discuss novel applications of neutron contrast variation in order to gain unique insights into the structure, dynamics, and molecular interactions of biological membranes. We specifically focus on how small-angle neutron scattering data is modeled using different contrast data and molecular dynamics simulations. We also briefly discuss neutron reflectometry and present a few recent advances that have taken place in neutron spin echo spectroscopy studies and the unique membrane mechanical data that can be derived from them, primarily due to new models used to fit the data.

## 1. Introduction

Membranes are critical structural and dynamic assemblies in biological cells, whose underlying structure is a lipid bilayer. In addition to acting as barriers separating cells and cell organelles from their external environments, cellular membranes perform a range of critical roles, such as the compartmentalization of cellular processes, hosting resident proteins, mediating cell signaling, and responding to environmental cues (Singer and Nicolson, 1972; Simons and Toomre, 2000; Phillips et al., 2009). In particular, the plasma membrane (PM), which performs the aforementioned role of enveloping cells and whose general structure was proposed in 1972 by Singer and Nicolson (Singer and Nicolson, 1972), has received a great deal of attention. Specifically, the PM differentiates itself from other cell membranes by being chemically asymmetric, where the chemical composition between its two bilayer leaflets is different (Verkleij et al., 1973). For example, saturated acyl chain phosphatidylcholines are predominantly located in the outer or exoplasmic leaflet, whereas phosphatidylserine (PS), phosphatidylethanolamine (PE), and phosphatidylinositol headgroup lipids, with varying degrees of unsaturation in their acyl chains, are located in the inner, cytoplasmic bilayer leaflet (Verkleij et al., 1973; Op den Kamp, 1979; Van Meer et al., 2008; Lorent et al., 2020). PM asymmetry is actively maintained by ATP driven processes that locate and maintain the individual lipid species in their respective bilayer leaflet, as lipids, if left to their own devices are able to spontaneously migrate between the two bilayer leaflets, a process known as lipid flip-flop (Seigneuret and Devaux, 1984; van Helvoort et al., 1996; Manno et al., 2002; Smith and Lambert, 2003). The breakdown of PM asymmetry can eventually lead to cell death or apoptosis (Williamson et al., 2001; Doktorova et al., 2020a).

Membranes are also dynamic structures, displaying a diversity of motions ranging from axial rotation of individual lipid molecules, to collective membrane fluctuations involving hundreds of lipids (Woodka et al., 2012). Since membrane dynamics are an integral component of the structure–function relationship, much effort has been expended in studying this relationship and different techniques have been brought to bear in trying to determine the structural and material properties of model membrane systems, including different neutron and x-ray scattering techniques (Wiener and White, 1992; Henriksen and Ipsen, 2004; Kučerka et al., 2008; Yi et al., 2009; Heberle et al., 2016).

Neutron scattering techniques have over the years evolved into unique and powerful nanoscopic probes of membrane structure and dynamics (Pfeiffer et al., 1993; Pan et al., 2013). Results from neutron scattering experiments have produced new insights, not only with regard to the material properties of membranes (e.g., bending rigidity), but also pertaining to their physical properties and how they relate to biological function. These insights have led to a better understanding of protein–lipid interactions, lipid domain formation and size, and PM asymmetry (Clifton et al., 2013; Marquardt et al., 2015; Heberle et al., 2016; Nickels et al., 2017; Rickeard et al., 2020).

Although less abundant than x-rays, neutrons have unique properties that make them ideal probes for the study of hydrogen-rich materials, such as biological membranes. One of those properties is that neutrons interact differently with protium, the most common isotope of hydrogen, and deuterium, the other stable hydrogen isotope (Svergun et al., 2013). This ability to differentiate between the isotopes of hydrogen enables a technique, commonly known as contrast variation, that permits one to highlight the membrane feature of interest. For example, by substituting normal water (H_2_O) for heavy water (D_2_O), the penetration depth of water into the membrane can be measured with a high degree of accuracy (Fitter et al., 1999). Although atomistic detail can be challenging, neutron scattering can reveal structures down to nanometer length scales, particularly when deuteration strategies are combined with robust membrane models and computer simulations, as was done in the case of the scattering density profile (SDP) model (Eicher et al., 2017). This combination allows neutron scattering to accurately determine bilayer thicknesses and the location of molecular species within the membrane (Kučerka et al., 2008; Marquardt et al., 2013, 2016). Further, deuterated lipids and proteins can be used to study lipid domain formation and protein–lipid interactions (Clifton et al., 2013).

The utility of neutron scattering not only lies in the structural determination of nanoscale membrane features, but also in the measurement of membrane dynamics that can be used to determine membrane mechanical properties using the collective motions of the membrane. Techniques, such as inelastic and quasielastic neutron scattering are sensitive to energy transfers commensurate with the energy of select dynamical modes, much of which remain poorly understood. Among these techniques, neutron spin echo (NSE) spectroscopy is sensitive to dynamical modes that take place over a range of length (~1–100 nm) and time scales (~1–1,000 ns), thus enabling studies of collective lipid dynamics and membrane fluctuations (Nagao, 2009). Similar to structural studies involving contrast variation, studies of membrane dynamics using NSE can similarly make use of contrast variation to tease out additional dynamic modes. Thus, the ability to adjust the contrast of specific chemical moieties within the membrane *via* deuteration enables different neutron scattering techniques to access structural and dynamical information that are not easily accessible by other commonly available methods.

In the first part of the review, studies that have made use of isotopic labeling to locate biomolecules, understand membrane asymmetry, and measure the bilayer thickness of the PM of a bacterium will be discussed. This will be followed by an overview of neutron contrast variation and how it has been applied to detect and understand nanoscopic lipid domains, including those in fully functioning bacteria. Later, a detailed description of the SDP model, whose derived structural parameters are widely used in different contexts, including to validate computer simulations of single component lipid bilayers will be presented. The review concludes with a summary of the NSE technique that was developed in the 1970s and is currently experiencing renewed interest in membrane research as a result of robust new models to analyze the experimental data.

## 2. Neutron Contrast Variation

One of the parameters used to quantify how neutrons interact with nuclei is the coherent scattering length, *a*_0_, which is related to the scattering cross section *via* $\sigma ≃4\pi a_{0}^{2}$; this parameter also depends on the interactions between the neutron spin and nuclear spin of the scatterers. Thus, the scattering length is negative when the pseudopotential for the nuclear interaction is attractive and positive when repulsive. Of significance to biology, the neutron scattering lengths of protium and deuterium have opposite signs; specifically, protium has a scattering length of −3.74 fm, while that of deuterium is + 6.67 fm (Svergun et al., 2013). Importantly, this difference in scattering length between the two stable isotopes of hydrogen can be used to great effect by altering the scattering contrast between the membrane and its surrounding, or locating biomolecules within the membrane through the use of deuterated analogs. However, some caution should be observed when making use of deuteration, as it can reduce lipid phase transition temperatures (Guard-Friar et al., 1985; Bryant et al., 2019) and adversely affect the viability of living organisms (Bild et al., 2004; Mosin and Ignatov, 2012). Nevertheless, when used methodically, deuteration is a powerful tool that can uncover unique structural and dynamical membrane features that can lead to new biological insights.

Because of the cost and limited availability of deuterated lipids, protiated lipids are commonly studied in the presence of D_2_O, where the scattering length density for H_2_O is −0.56 × 10^−6^ Å^2^, while that of D_2_O is +6.35 × 10^−6^ Å^2^ (Clifton et al., 2013). Replacing H_2_O by D_2_O alters the contrast between the water and the membrane, with maximum contrast for a protiated membrane nominally achieved at 100% D_2_O. In addition to deuterated water, other components, such as polymers, detergents, and osmolytes can be deuterated to impart maximum contrast between the various components (Naumann et al., 1994; Toppozini et al., 2012; Oliver et al., 2017). The ability to contrast match the coherent neutron scattering of specific molecules or moieties, by matching their scattering length densities to the scattering length density of the buffer in which they reside in distinguishes neutron scattering from other scattering methods, such as x-ray and light scattering. This “probe-free” feature of neutrons thus enables a wide range of physicochemical studies that are nominally not accessible to other techniques (Knoll et al., 1981; Kučerka et al., 2009a; Clifton et al., 2013; Oliver et al., 2017).

### 2.1. Cholesterol's Location in Membranes

Cholesterol is an essential biomolecule of animal cell membranes and is closely associated with functional lipid domains, commonly referred to as lipid rafts. Cholesterol is also known to modulate membrane structure by altering its fluidity, thickness, and water penetration (Yang et al., 2016), properties that are most likely associated with the location of the sterol in the membrane. Despite cholesterol's importance in biology, its location in membranes is not as simple as was once thought and, as a result, has been the subject of much recent research.

**Figure 1:**
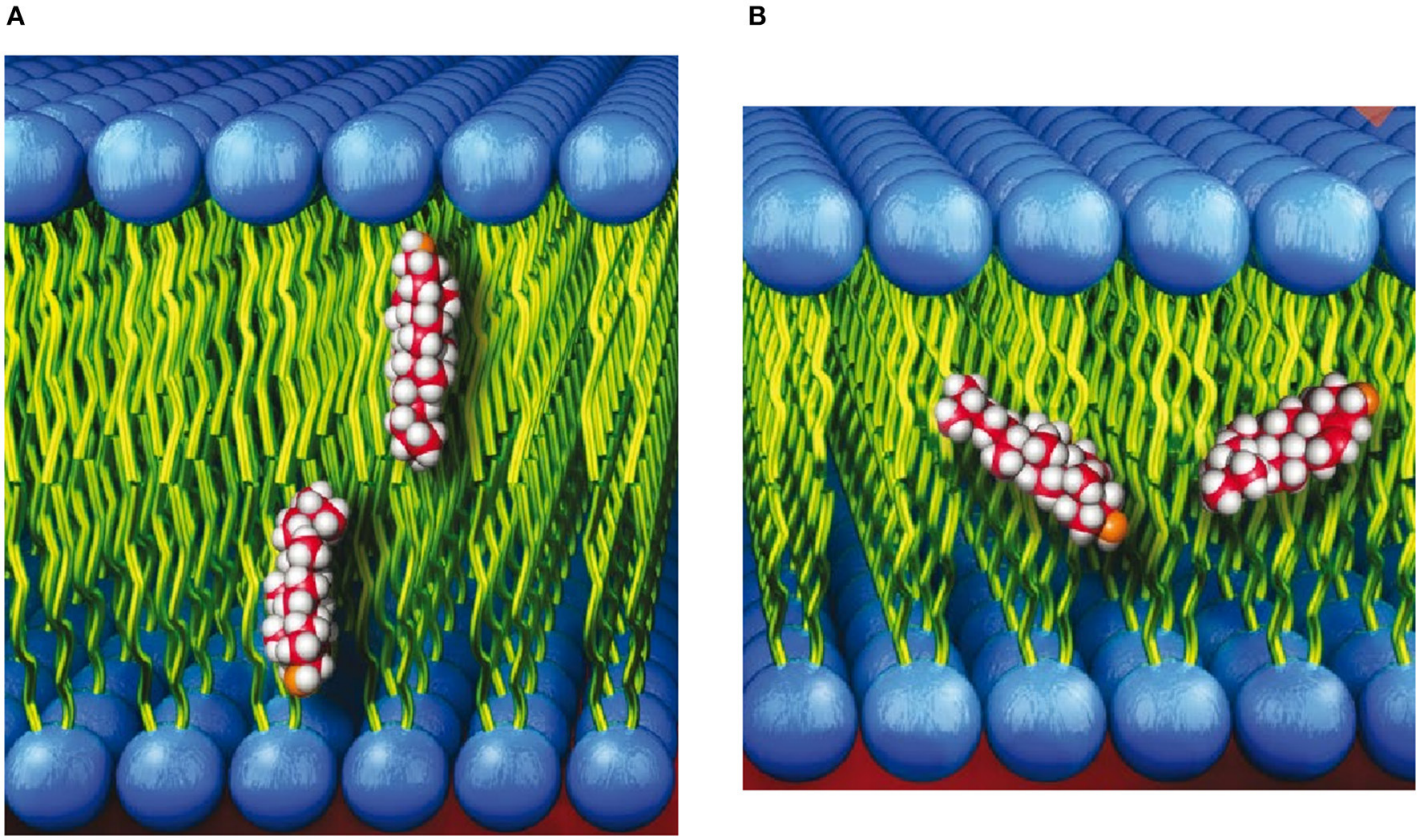
**(A)** Cholesterol shown in its commonly accepted upright orientation with its hydroxyl group near the lipid-water interface. **(B)** Cholesterol shown spanning the bilayer leaflets of membranes with “thin” hydrophobic cores.

In 2001, Léonard et al., using 14:0 phosphatidylcholine (PC) multibilayer stacks and deuterated or protiated cholesterol, carried out neutron diffraction studies on aligned multibilayers that showed the sterol oriented in its nominal upright position with its hydroxyl group near the lipid-water interface (Figure 1A) (Léonard et al., 2001). The neutron diffraction results by Léonard et al. were supported by MD simulations that were used to reconstruct the one-dimensional neutron SDP that was then compared to the one obtained from experiment.

The neutron scattering studies by Léonard et al. (2001) were subsequently followed by several studies detailing the location of cholesterol in lipid bilayers with saturated and polyunsaturated fatty acid (PUFA) chains (Harroun et al., 2006, 2008; Marrink et al., 2008; Marquardt et al., 2016). In contrast to bilayers with saturated acyl chains, neutron scattering, nuclear magnetic resonance (NMR), and MD simulations found that the location of cholesterol can deviate from its upright orientation (Figure 1A), to one where the sterol snorkels toward the middle of the bilayer (Figure 1B). Specifically, the study by Marquardt et al. (2016) that made use of tail and head group deuterated cholesterol and a series of PC lipids with different acyl chain lengths and degrees of unsaturation determined that cholesterol reorients rapidly about the bilayer normal in all PC membranes studied. However, in bilayers with a very thin hydrophobic core (i.e., bilayers with short, saturated fatty acid chains and those with PUFA chains), the sterol is tilted and forced to span the bilayer midplane (Figure 1B). Marquardt et al. also noted that the highly disordered nature of PUFA chains allows them to interact differently with cholesterol than with other types of fatty acids. In general, neutron scattering techniques have provided vital insight on how lipid species can alter the location of cholesterol.

To add nuance to the location of cholesterol in membranes, neutron scattering experiments have also provided insight into cholesterol transport in membranes. For example, Garg et al. (2011) used time-resolved small-angle neutron scattering to observe intermembrane exchange and intramembrane flipping rates of cholesterol. They discovered that other techniques, which may use tags or other compounds to facilitate such measurements, can greatly accelerate cholesterol exchange and flipping rates. As a result of the different neutron scattering studies, we currently have a better understanding of the location of cholesterol in biological membranes, as well as its transport in them.

### 2.2. Biomolecular-Membrane Interactions

**Figure 2:**
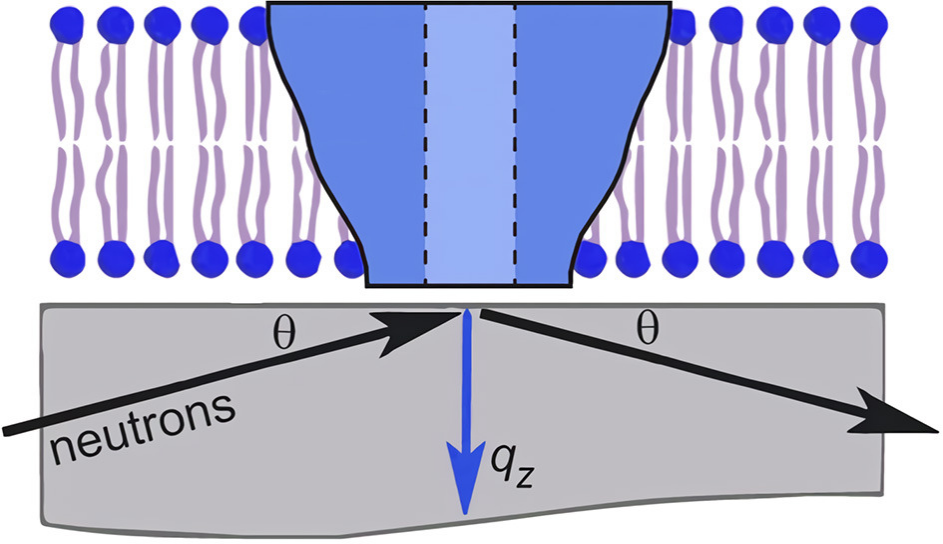
Schematic of an neutron reflectometry (NR) experiment involving a single lipid bilayer with an integral protein adsorbed to a solid support (usually single crystal Si). An incident beam of neutrons is transmitted through the substrate and is reflected from the different interfaces of the sample. The scattering vector, *q*_*z*_, is denoted by the blue arrow. The detection and analysis of the reflected beam allows for the determination of the one-dimensional SLD along the bilayer normal. Figure was adapted from Soranzo et al. (2017).

Neutron reflectometry (NR) is a powerful technique for determining the nanoscale structure of model membranes. For NR, membranes of interest are deposited either as bilayers or monolayers on solid substrates (e.g., single crystal Si) or as monolayers at the liquid–air interface. In contrast to scattering methods used to study powder or isotropic samples, NR makes use of a shallow incident beam to interact with the sample. The scattered intensity using NR geometry is then used to determine the one-dimensional SLD along the bilayer normal with nanoscale resolution. In the case of membranes, this includes membrane thickness, membrane asymmetry, and the location of molecules interacting with the membrane (Wong et al., 1999; Richter et al., 2006; Heinrich and Lösche, 2014; Heinrich, 2016; Campbell, 2018; Kurniawan et al., 2018). Figure 2 is a schematic of such an experiment, where Soranzo et al. used NR to study the conformation of the hepatitis C virus p7 protein in a lipid membrane and its effect on membrane structure (Soranzo et al., 2017).

A recent highlight of NR's capability to shed light on biomolecular-membrane interaction involved α-synuclein, a protein whose function is currently unknown, but abnormal deposits of the protein are found in the brain. These protein deposits are associated with Lewy body dementia (LBD), a disease that can lead to changes in mood, behavior, and motor skills. Recently, Pfefferkorn et al. (2012) used NR and computer simulations to determine the depth at which α-synuclein resides in lipid membranes and its associated membrane thinning effect. Further, Yap et al. (2015) studied glucocerebrosidase and α-synuclein interactions at the membrane surface using NR and fluorescence – it is believed that mutations of glucocerebrosidase increase the risk factors of Parkinson's disease. Using deuterated α-synuclein and protiated glucocerebrosidase, Yap et al. showed a large change in the membrane-bound structure of α-synuclein. Importantly, they proposed a model of α-synuclein and glucocerebrosidase for interacting at the membrane, where glucocerebrosidase is inhibited by α-synuclein, displacing glucocerebrosidase away from the membrane and perturbing the active site. In doing so, glucocerebrosidase alters the structure of α-synuclein, impacting its degradation. According to Yap et al. (2015), these events can lead to the development of Parkinson's disease. In another NR study, Jiang et al. (2017) used native chemical ligation and segmental deuteration to determine the regions of α-synuclein that bind to cellular membranes, and in 2019, Perissinotto combined NR, NSE, and atomic force microscopy to show that GM1 ganglioside can act as a membrane-binding mediator for α-synuclein (Perissinotto et al., 2019). The series of NR α-synuclein studies recounted in this review, is only one example among many demonstrating the impact that NR can have on membrane-related research of α-synuclein (Hellstrand et al., 2013; Jiang et al., 2015; Kaur and Lee, 2021).

Although α-synuclein was used as a primary example to highlight how NR can be used to elucidate biomolecular-membrane interactions, the utility of NR extends far beyond this protein. For example, NR has been used in the study of prions (Le Brun et al., 2014), synthetic peptides (Smith et al., 2010), Alzheimer's disease (Rondelli et al., 2016), toxins (Chenal et al., 2009; Wacklin et al., 2016; Sani et al., 2020), and ion channels (Holt et al., 2005, 2009; Rondelli et al., 2018). Of note, in the past decade, the field of biomolecular-membrane interactions has grown significantly, highlighting the utility of NR as an essential tool in the study of surface interactions in biology.

### 2.3. Membrane Hydrophobic Thickness of Viable Bacteria

**Figure 3:**
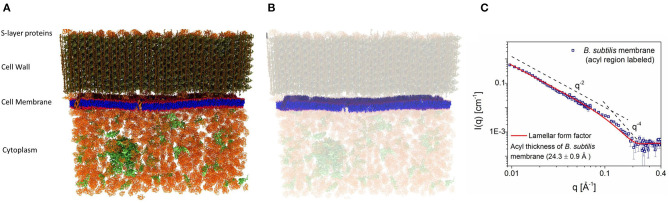
**(A)** Schematics of the wild type *Bacillus subtilis* cell wall, membrane, and cytoplasm. **(B)** Bacteria grown in deuterated media, replacing its protium for deuterium, were fed protiated fatty acids to introduce neutron contrast. Under these conditions, the protium-labeled membrane is the only structure visible to neutrons when the bacteria were immersed in 85% heavy water (D_2_O). **(C)** Small angle neutron scattering (SANS) data from the system in B were fitted using a lamellar form factor, resulting in an average hydrophobic membrane thickness of 24.3 ± 0.9 Å. Figure adapted from Nickels et al. (2017).

Because of their inherent complexity, viable cells are rarely used to study their nanoscale structures and, for the most part, researchers rely on model systems to provide nanoscopic information to infer biological function. Nickels et al. (2017) used a novel approach to allow for the determination of detailed membrane structure in the Gram-positive bacterium, *Bacillus subtilis*. Through genetic and chemical manipulations, Nickels et al. were able to label the bacterium and its membrane independently with deuterium and protium, respectively, enabling them to determine the average hydrophobic thickness (24.3 ± 0.9 Å) of the bacterium's PM (Figure 3). Specifically, most of the protium of *B. subtilis* was replaced with deuterium, making the entire organism invisible to neutrons when immersed in ≈ 85% D_2_O. With its fatty acid synthesis and degradation pathways shutdown, the bacteria were fed protiated fatty acids to introduce neutron contrast (Figure 3B) and were then subjected to experimentation. Figure 3C is the first direct SANS measurement of the hydrophobic thickness of a PM in a viable organism.

### 2.4. Plasma Membrane Asymmetry

**Figure 4:**
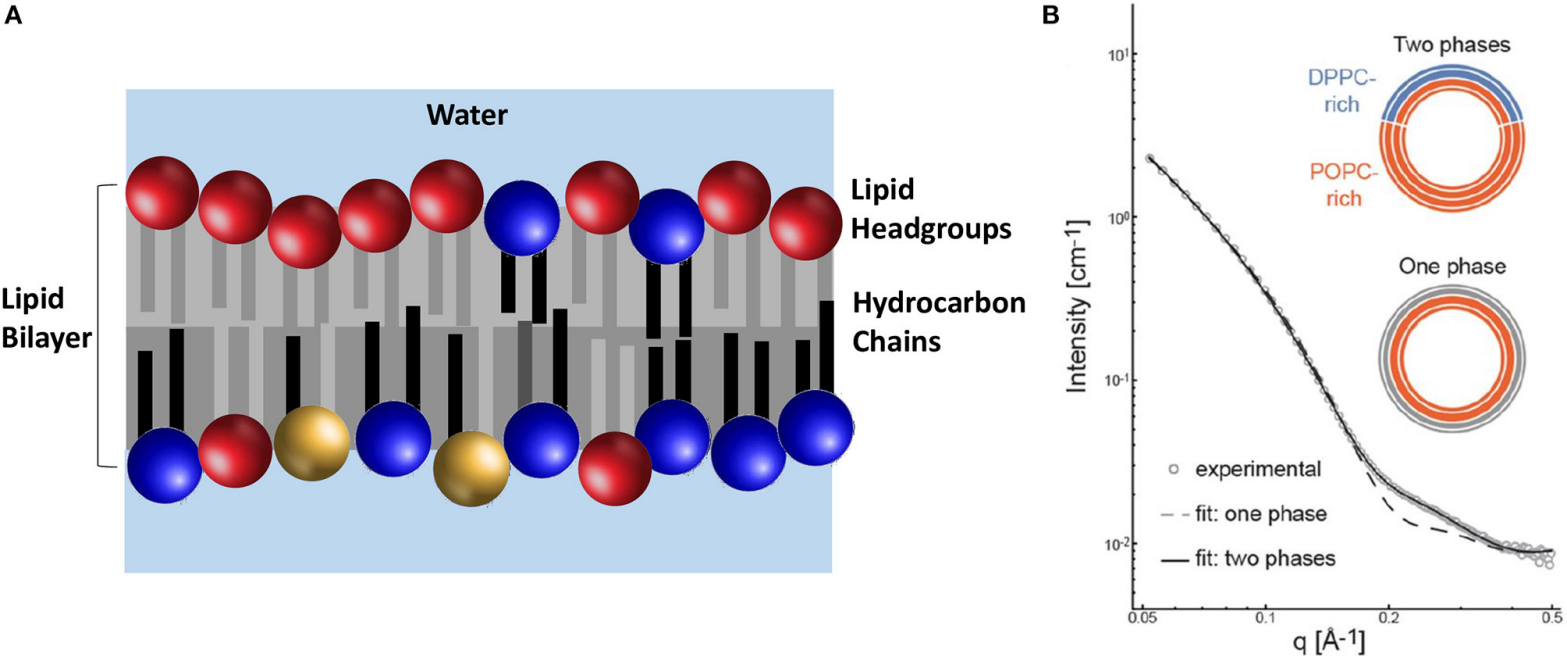
The lipid composition of the PM is asymmetric with regard to the location of its chemically different lipid molecules. **(A)** The outer (upper) leaflet contains different lipid headgroups (primarily PC headgroups lipids shown in red) in relation to the inner (lower) leaflet (primarily blue and yellow depicting PE and PS headgroups, respectively). Additionally, the composition of the acyl chains between the two leaflets is also different. The outer leaflet has lipids with mostly saturated acyl chains (gray), whereas the inner leaflet lipids have acyl chains with different levels of unsaturation (black and light gray). **(B)** SANS data of aULVs where the outer leaflet contains 34 mol% of a rigid saturated lipid (DPPC) and the inner leaflet contains 98 mol% of a fluid state unsaturated lipid (POPC). The best fit to the data was obtained using a model that took in to account the presence of two lipid phases in the outer leaflet. Importantly, DPPC was found to be more fluid, compared to a pure DPPC bilayer, suggesting an interaction between the two bilayer leaflets. Adapted from Heberle et al. (2016).

It should not come as a surprise that biology has evolved to create membranes with great lipid diversity that impart to it remarkable functionality. In addition to being laterally heterogeneous (see page 10), the PM is also asymmetric. In other words, the lipids found in the inner and outer leaflets of the bilayer are chemically different (Figure 4A). However, many biophysical studies investigating the PM have not included this feature. Therefore, even though we have known that lipid diversity and lipid asymmetry are natural features of biological membranes (Verkleij et al., 1973), symmetric lipid bilayer systems have been used to study the physical properties of membranes. This is due, to a great extent, to the fact that the techniques needed to produce stable PM mimetics have proven to be difficult to implement and have produced mixed results.

Recently, a method was developed to reproducibly generate stable asymmetric unilamellar vesicles (aULVs) through the use of cyclodextrin carrier molecules (Cheng and London, 2009). Since the production of aULVs using this technique uses aggressive cyclodextrin and centrifugal filtration, possibly adversely affecting the membranes, Heberle et al. (2016) prepared isotopically asymmetric 16:0/18:1 PC (POPC) aULVs, where the 16:0 chain and headgroup of the lipid were deuterated, in order to determine any adverse effects to the membrane as a result of the method used to prepare them. From the fits to their SANS data, Heberle et al. determined that isotopically asymmetric POPC vesicles had the same bilayer thickness and area per lipid (AL) as symmetric POPC ULVs. In addition, again using deuterated lipids, they partially exchanged 16:0/16:0 PC (DPPC) into the outer bilayer leaflet of POPC ULVs, producing aULVs with chemical asymmetry. At 20°C, the outer bilayer leaflet was phase separated (i.e., gel DPPC and fluid POPC), while the POPC-rich inner leaflet was fluid (Figure 4B). Using this experimental approach, Heberle et al. (2016) were able to resolve structural details of the individual bilayer leaflets and discovered that the fluid POPC-rich was able to partially “fluidize” the gel-like outer leaflet, implying the presence of interleaflet coupling and the possibility that bilayer asymmetry may be an integral part of lateral membrane organization.

Following the studies by Heberle et al. (2016), additional neutron scattering studies have appeared reporting on membrane asymmetry. The asymmetric DPPC/POPC membrane system studied by Heberle et al. at 20°C was revisited by Eicher et al. (2017) They reported that, at 50°C, where both bilayer leaflets are in the fluid phase – above the melting transition of both lipids – there was no evidence of transbilayer coupling between the inner and outer bilayer leaflets. In other studies, membrane asymmetry was used to report on lipid flip-flop rates in pure lipid aULVs (Nguyen et al., 2019) and in aULVs with membrane associated peptides (Doktorova et al., 2019a; Nguyen et al., 2019). For example, in pure lipid systems the lipid flip-flop rates in a closed vesicle system were much slower than previously reported values from supported asymmetric bilayer systems (Liu and Conboy, 2005; Marquardt et al., 2017; Nguyen et al., 2019). Additionally, lipid flip-flop rates were shown to be influenced by the presence of methanol as well as, membrane peptides, suggesting that membrane defects are a contributing factor to lipid flip-flop (Doktorova et al., 2019a; Nguyen et al., 2019).

### 2.5. Detecting Lipid Domains in Model Membranes

The lateral organization of biological membranes seems to play a substantial role in biology and its existence has been debated since the unveiling of the fluid mosaic model in 1972 (Singer and Nicolson, 1972) – certainly since the development of the raft hypothesis (Simons and Ikonen, 1997; Kinnun et al., 2020). In fact, lipid domains have been implicated in membrane processes such as, vesicular transport, protein sorting, and cell signaling, to name just a few. Early on, it was determined that model membranes whose lipids were homogeneously distributed could be contrast matched, but those with inclusions or heterogeneities (e.g., domains) could not (Knoll et al., 1981). This is due to the fact that the domains have a different SLD than the bulk membrane in which they reside. However, by varying the percent D_2_O to contrast match the bulk membrane, the domains become “visible” to neutrons and one can isolate their scattering signal. Taking this one step further by using a mixture of deuterated and protiated lipids that colocate within the membrane domain, a scattering signal with an improved signal-to-noise ratio can be obtained (Pencer et al., 2005; Masui et al., 2008).

**Figure 5:**
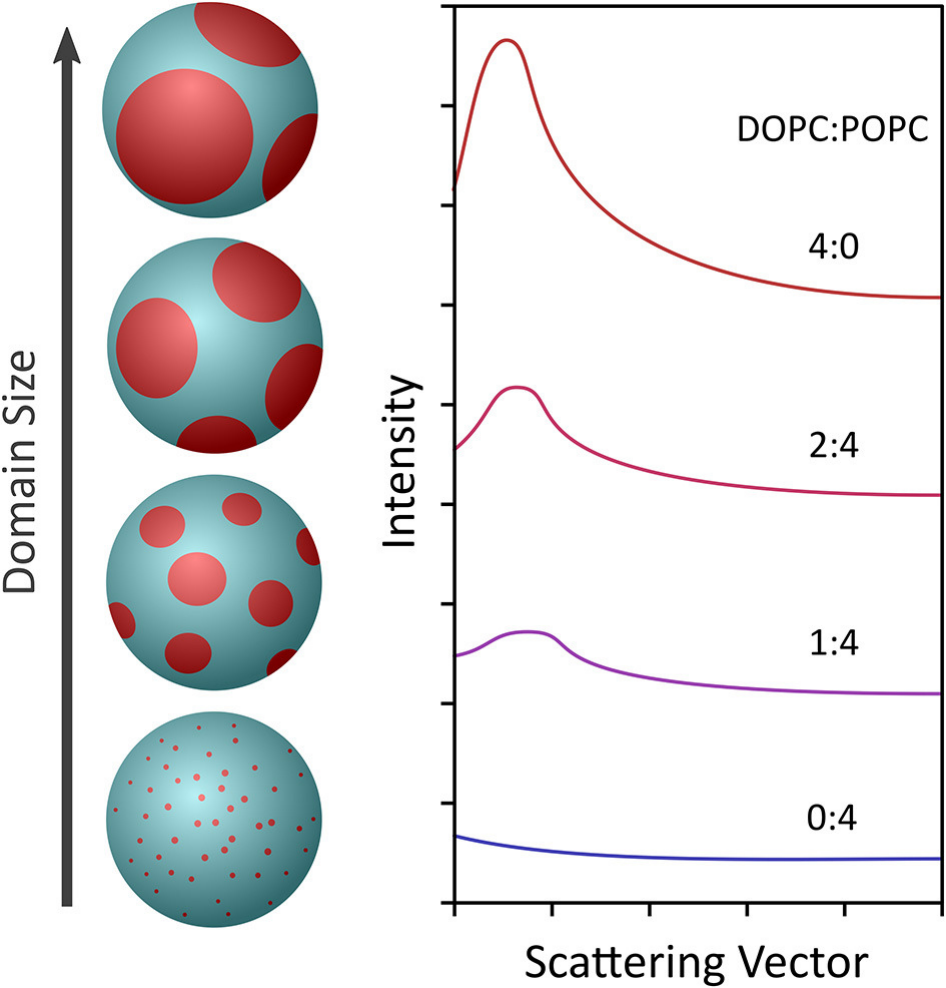
Schematic showing the use of contrast variation to observe the formation of nanoscopic lipid domains in 60 nm diameter ULVs (left) made from a lipid composition (i.e., DSPC/DOPC/POPC/cholesterol) mimicking the outer leaflet of a mammalian PM. At a DOPC:POPC ratio of 0:4, the domains were extremely small, producing minimal scattering (right). However, as the amount of DOPC with respect to POPC increased, the domains grew larger and the scattering signal increased. The scattering data were fitted using Monte Carlo simulations, which gave an indication of the diameter and number of nanoscopic domains in a ULV of given lipid composition. Figure adapted from Heberle et al. (2013).

This approach was demonstrated by Heberle et al. using a 60-nm diameter unilamellar ULVs composed of a four-component phase separating lipid mixture with fixed amounts of cholesterol and a chain deuterated phospholipid (DSPC), and varying amounts of monosaturated (POPC) and/or diunsaturated (DOPC) lipids (Heberle et al., 2013). Since the detection of domains requires that they have an SLD different from the coexisting bulk phase, chain deuterated DSPC was used. Using this approach, Heberle et al. were able to observe the presence of nanoscopic lipid domains whose size increased with increasing amounts of DOPC, as shown in Figure 5. Further, bilayer thickness mismatch between the domains and the bulk membrane also increased with the increasing domain size, in agreement with line tension, theories and the first experimental verification of said theories in free-floating bilayers. Moreover, the results by Heberle et al. suggested a mechanism by which the size of functional domains in homeothermic organisms may be regulated through changes in lipid composition. The experimental approach by Heberle et al. (2013) was subsequently extended by Nickels et al. to study the mechanical properties of membranes and their lipid domains by NSE (Nickels et al., 2015). Using a combination of deuterated lipids and different solvent deuteration schemes, Nickels et al. were able to highlight or mute the nanoscopic lipid domains populating 60 nm diameter ULVs.

### 2.6. Lipid Domains in Viable Bacteria

As mentioned in the previous section, because of their inherent complexity viable cells are rarely used in scattering studies of nanoscale membrane structures. As such, we have relied extensively on the use of model membrane systems to gain biological insights. However, model membranes are often simplified in composition and structure, and always lack the active processes found in cells. One of the pressing questions in cell biology is whether living cell membranes also exhibit nanoscopic lipid organization, i.e., lipid domains that are routinely observed in model membrane systems. It is now widely accepted that lipid domains are nanoscopic, as well as transient, making them difficult to detect experimentally (Mukherjee and Maxfield, 2004; Lingwood and Simons, 2010). *In vivo* membrane studies at lower resolution have provided details of lateral membrane structure, as well as important information pertaining to dynamic processes (Eggeling et al., 2009).

**Figure 6:**
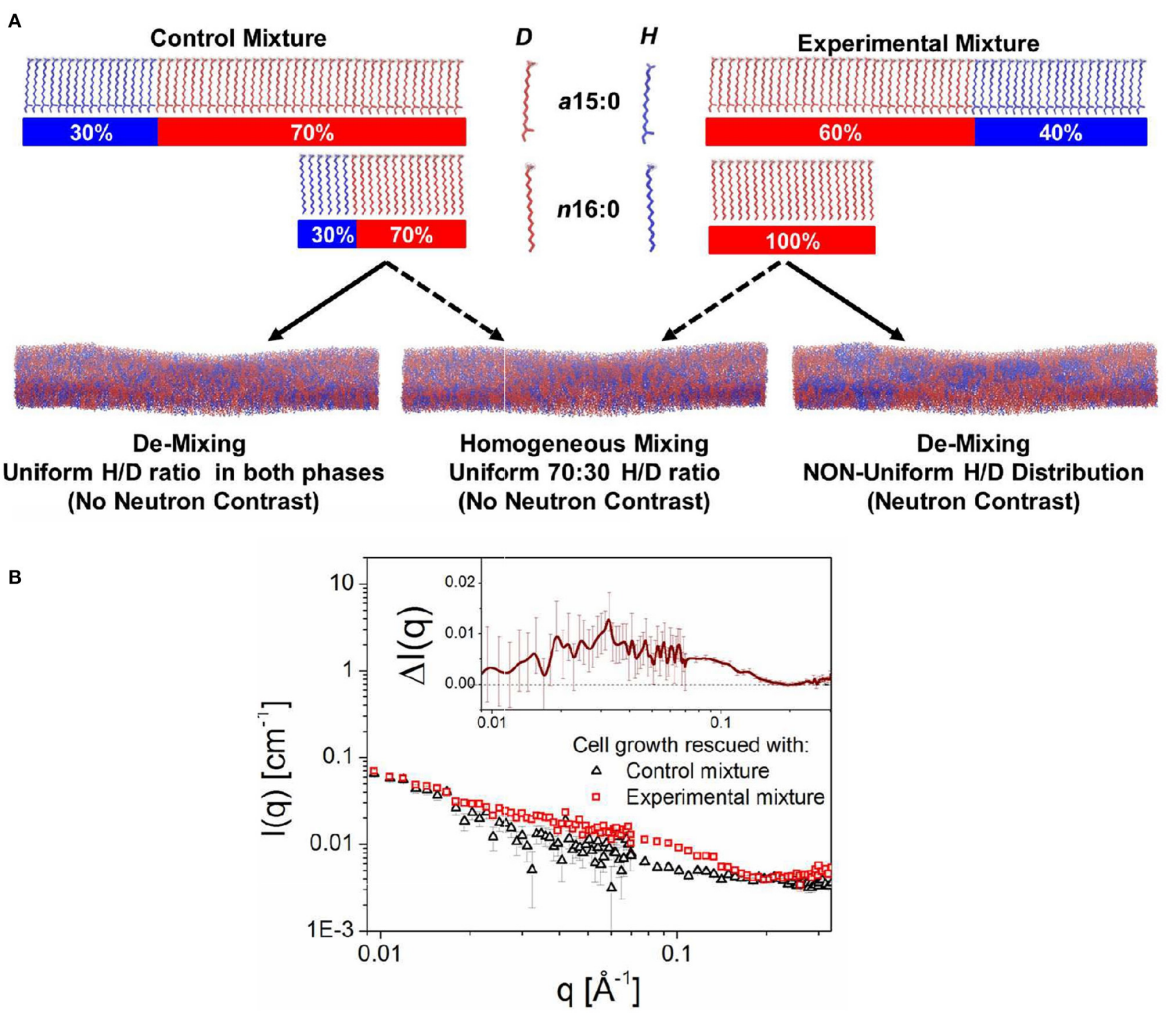
Schematic showing the approach that was used to detect lipid domains in the PM of *B. subtilis*. The bacteria were genetically and chemically manipulated to contain only two fatty acids, namely the low temperature melting a15:0 and the high temperature melting n16:0. Note, that *B. subtilis* incorporates a15:0 at more than twice the amount of n16:0. **(A)** The control fatty acid mixture consisted of a15:0 and n16:0, each of ratio 30% protiated:70% deuterated. On the other hand, the experimental fatty acid mixture consisted of an isotopic distribution of 40:60 protiated:deuterated for a15:0 and 100% deuteration for n16:0. **(B)** Scattering curves from bacteria with control and experimental fatty acid mixtures. The excess scattering is shown in the inset to the figure. Figure adapted from Nickels et al. (2017).

Recently, Nickels et al. (2017) were able to use genetic and chemical manipulations to label a live bacterium and its membrane with deuterium and protium, respectively, enabling them to determine the average hydrophobic thickness of the bacterium's PM (see Figure 6). Their next step was to determine whether or not lipid domains existed in *B. subtilis'* PM. Since they could not use the approach of Heberle et al. (2013), where the homogeneously mixed membranes at an elevated temperature were contrast matched to the solvent and their presence detected once the lipids phase separated at low temperature, Nickels et al. developed a novel approach of detecting lipid domains in a living organism.

The approach by Nickels et al. (2017) consisted of growing cells with two different ratios of anteiso 15:0 (a15:0) and normal 16:0 (n16:0) fatty acids, the only fatty acids present in the membrane of the genetically manipulated bacteria in protiated/deuterated ratios such that the two mixtures had, on average, the same scattering lengths and could be contrast matched to 85% D_2_O. The “control” fatty acid mixture consisted of 15:0 (low melting) and n16:0 (high melting) fatty acids, each 30% protiated and 70% deuterated (Figure 6A). The SANS curve for this fatty acid mixture is shown in Figure 6B. The so-called “experimental” mixture contained the same fatty acids, but with an isotopic distribution for a15:0 of 40:60 protiated:deuterated, and 100% deuterated n16:0. Since the neutron scattering lengths of these two fatty acid mixtures are the same, and if the membranes lacked lateral heterogenity, the SANS patterns from both the control and experimental mixtures would be the same. However, as can be observed from Figure 6B, this was not the case, implying that the PM of *B. subtilis* is populated with lipid domains specifically, domains ranging in size between 3 and 40 nm (Nickels et al., 2017).

The observation of nanoscopic lipid domains in the PM of *B. subtilis* is consistent with the notion of lipid rafts. This experimental approach developed by Nickels et al. (2017) allows for a wide range of structural studies of the cell membrane (and possibly other classes of biomolecules) without the need for bulky extrinsic probes or labels. In this way, it fundamentally changes the scope of nanoscale structural questions that can be addressed in living organisms.

### 2.7. The SDP Model

A tenet in biology is that structure enables function. Structure–function relationships have arisen over the millennia through processes of natural selection and in order to understand how individual or ensembles of biomolecules function in biology, it is necessary to obtain accurate structural and dynamical data. In general, protein crystallography has resulted in excellent structures of predominantly water soluble proteins (Wiener and White, 1991a), but this holds less true for membranes and membrane associated proteins, partly because they are highly heterogeneous, disordered assemblies, whose structure–function relationships begin to emerge only with ensembles of hundreds of cooperating molecules and at length scales greater than about 10 nm (Wiener and White, 1991a; Nagle and Tristram-Nagle, 2000; Heberle et al., 2012). This generally makes membranes unsuitable for study by traditional structural biology methods (e.g., crystallography) that have revolutionized the study of water soluble proteins and nucleic acids.

Neutron scattering data has been analyzed using different models of the bilayer. The most basic of these models are the so-called strip models. In a strip model, the membrane is subdivided, not surprisingly, into strips of given SLD, with the simplest strip models treating the bilayer as one single strip of constant SLD (Knoll et al., 1981; Komura et al., 1982; Nawroth et al., 1989; Mason et al., 1999; Pencer and Hallett, 2000). Refinements include additional strips with different SLDs (Balgavỳ et al., 2001; Schmiedel et al., 2001). Strips can represent, for example, lipid headgroups, hydrocarbon chains, and acyl chain terminal methyl groups. Additional geometric considerations, such as bilayer asymmetry not only improve the fits to the data but can also introduce a problem namely, that the system cannot have more parameters than observations or the system becomes over parameterized (Kučerka et al., 2004). However, this problem can be mitigated when different neutron contrast scattering data are combined with x-ray scattering – providing yet another contrast – and MD simulations.

Besides strip or slab models (King and White, 1986; Kučerka et al., 2004; Kiselev et al., 2006; Pencer et al., 2006), other models used over the years to analyze the structure of membranes and their individual lipid molecules were so-called Gaussian models (Kučerka et al., 2004, 2007; Kiselev et al., 2006; Pencer et al., 2006) and hybrid models (combination of Gaussian and strip/slab models) (Wiener et al., 1989). However, all these methods had one thing in common, an inability to jointly analyze different contrast data sets. Since neutrons are inherently sensitive to regions of the lipid membrane lacking in naturally abundant hydrogen (i.e., acyl chain carbonyl groups) and x-rays are uniquely sensitive to regions of high electron density (e.g., the phosphate groups in lipid head groups), having the ability to jointly analyze both types of different contrast data with a single model greatly increases the robustness of the proposed structural solution (Wiener and White, 1992). To address this problem, Wiener and White developed the composition space model (CSM) that enabled, for the first time, the joint analysis of neutron and x-ray lipid bilayer datasets (Wiener and White, 1991b).

CSM was based on the approach, initially developed by King and White, invoking a quasimolecular model, where the different moieties of a lipid molecule are described by Gaussians, thereby reducing the amount of parameters needed to accurately model the membrane (King and White, 1986). CSM was expanded on the model of King and White by including x-ray data to the neutron data, thus increasing the number of observations relative to the number of parameters used in the model – as mentioned, membranes are inherently under determined systems. In the CSM method of analysis, this inclusion was achieved by devising a parsing scheme for the lipid molecule that aligned the neutron and x-ray centers-of-scattering using atomic coordinates from lipid crystal structures projected onto the bilayer normal (Wiener and White, 1991b). Once the parsing scheme is defined, the modeling is carried out by scaling the time-averaged distributions of the different Gaussians describing the bilayer, using either neutron or x-ray scattering lengths, eventually giving rise to membrane structural parameters (Wiener and White, 1991b). Although this approach was highly successful, there were also drawbacks. First, the parsing scheme was, for the most part, based on trial and error (Wiener and White, 1991b, 1992). Second, CSM made use of structure factors from aligned membrane stacks hydrated to only 66% relative humidity (RH), a level of hydration that led to better quality data, as membranes are more ordered when not fully hydrated, thus resulting in more orders of Bragg diffraction, i.e., higher resolution data (Wiener and White, 1992). Third, and as pointed out by Armen et al. (1998), CSM allowed for RMS variability in the local volume of 7%.

**Figure 7:**
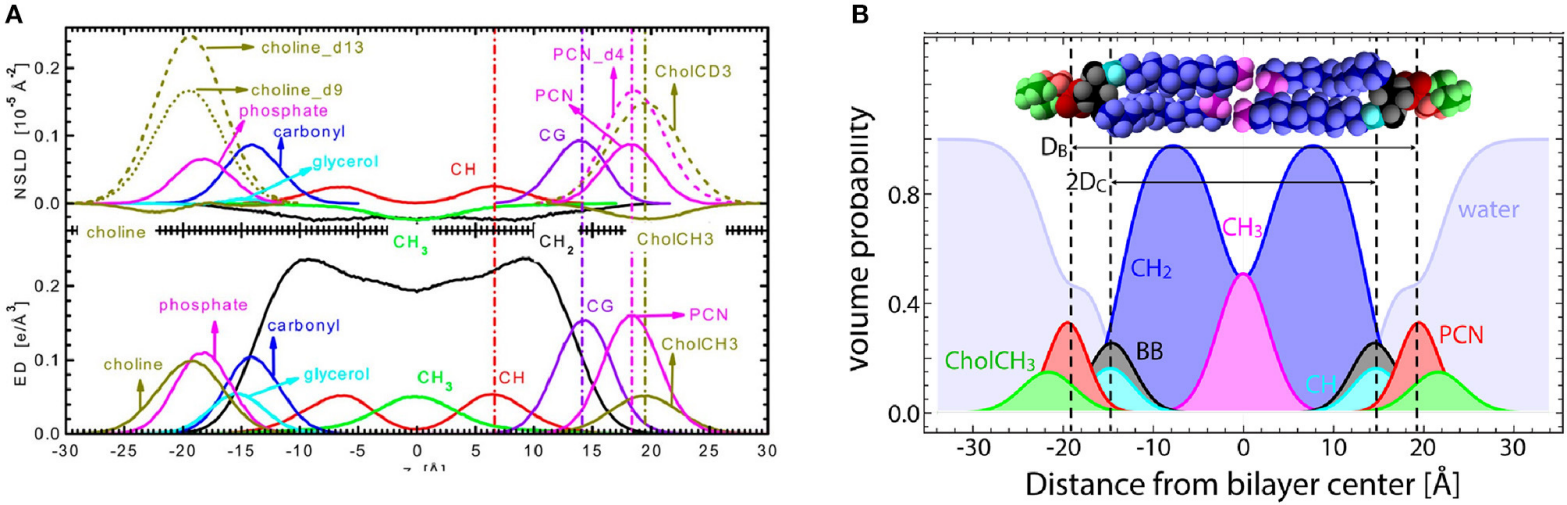
The SDP model. **(A)** Neutron SLD (NSLD) and electron density (ED) profiles obtained from MD simulations as a function of distance (z) along the bilayer normal of a PC bilayer. The left-hand side of the panel shows the individual components of a PC lipid molecule, whereas the right-hand side shows the appropriately parsed component groups. Dashed vertical lines indicate the peak location in the NSLD (upper) and ED (lower) profiles. For the parsing scheme used in the SDP model, component grouping peak profiles align for both NSLD and ED profiles, even when certain component groups are deuterated, as is often done in neutron scattering. **(B)** Volume probability distributions for the parsed component groups as a function of z along the bilayer normal used in the structural determination of sphingomyelin bilayers. Volume probability distributions are used to model raw scattering form factors by calculating ED or NSLD profiles and then Fourier transforming them to fit the raw scattering data. Figure was adapted from Kučerka et al. (2008) and Doktorova et al. (2020a).

Kučerka et al. (2008) introduced the SDP model that made use of continuous form factors obtained from small angle scattering experiments. In a similar spirit, Shekhar et al. (2011) developed the continuous distribution model that utilized error functions as opposed to Gaussian distributions in 2011. The SDP model relies on the use of different contrast neutron and x-ray data from ULVs under physiologically relevant conditions (i.e., vesicles in bulk water, a condition that is equivalent to hydration under 100% RH conditions) (Katsaras, 1997, 1998). Importantly, and in contrast to CSM, MD simulations were used to optimize the parsing of lipid molecules, an integral and novel part of the SDP model. In addition, SLDs were obtained by scaling the volume distributions of the individual lipid moieties with the appropriate neutron or x-ray scattering lengths (Figure 7) (Kučerka et al., 2008). The SDP also allowed for water to penetrate any voids in the bilayer, such that the volume probability distributions along the bilayer normal always added up to unity.

Although the data from ULVs does not extend as far out in reciprocal space as data from aligned multibilayer lipid stacks, the combined use of lipid parsing schemes determined by MD simulations, the inclusion of independent volumetric data, the notion of volume probability distributions, and the use of fully hydrated samples with different contrast data have enabled SDP to consistently produce robust structural results. For example, one of the most important lipid bilayer structural parameters is area per lipid (AL). AL is used to understand bilayer structure and gain insights into lipid–lipid and lipid–protein interactions (Wiener and White, 1992; Kučerka et al., 2008). Moreover, AL allows for comparisons between different lipid types and is central to MD simulations. In fact, MD force fields are considered to be accurate if they are able to reproduce experimentally determined lipid areas. ALs derived from SDP model analysis are now widely used to validate experimental approaches, including MD force fields (Lee et al., 2014; Doktorova et al., 2017; Grote and Lyubartsev, 2020).

To date, the SDP model has been successfully used to determine the structure of different membranes, including PC (Kučerka et al., 2008, 2009b, 2011; Marquardt et al., 2020), phosphatidylglycerol (PG) (Kučerka et al., 2012; Pan et al., 2014a), PS (Pan et al., 2014b), PE (Kučerka et al., 2015), and sphingomyelin (Doktorova et al., 2020b) lipids. Many of these studies focused on a single head group species, while sweeping through different acyl chains (both in length and degree of unsaturation). From these studies, the idea that lipid head groups control lipid packing, while the acyl chains dictate the response to changes in temperature, has been, for the most part, validated (Kučerka et al., 2015). Additionally, it has been shown that exchangeable hydrogens should be taken into consideration as they can alter the overall scattering signal (Pan et al., 2014a). One clear conclusion from the development of the SDP model is that the understanding of the structure of individual lipid molecules has been both changed and enhanced since its development. As more structures are determined, they will enable us to better understand the structure–function relationship in cellular membranes.

## 3. Neutron Spin Echo

**Figure 8:**
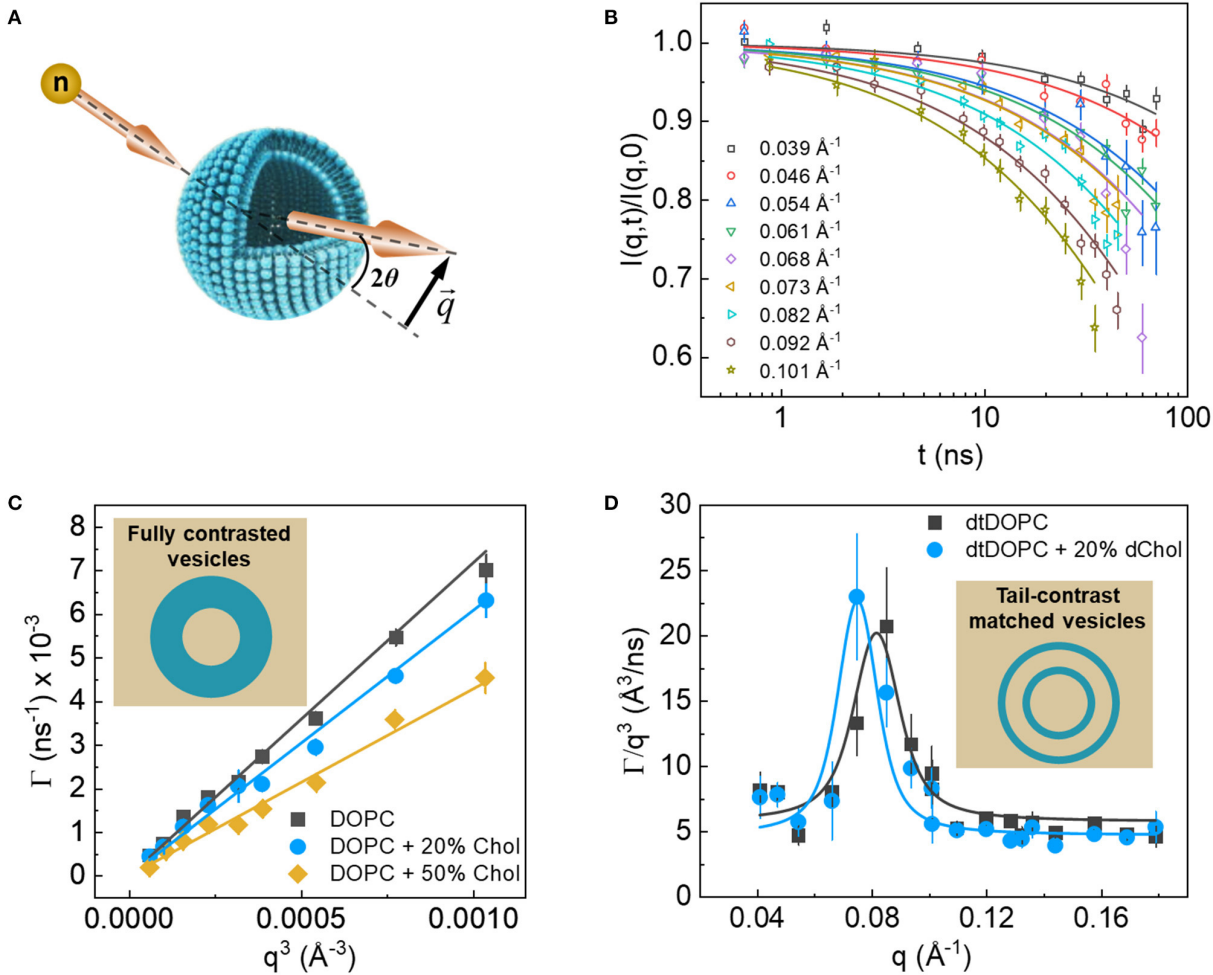
**(A)** Schematic of the scattering geometry used in NSE experiments. **(B)** Example of the NSE intermediate dynamic structure factors resulting from ULVs. **(C)** Plot of the *q*^3^ dependence of fluctuation decay rates of protiated membranes in D_2_O (inset), where shallower slopes indicate stiffer membranes according to the Zilman-Granek theory. **(D)** Excess dynamics, typical of thickness fluctuation signals from tail contrast matched membranes (inset). Data are adapted from Chakraborty et al. (Figures 2, 5).

Neutron spin echo spectroscopy (Figure 8A) offers a unique approach for direct quantitative measurements of collective membrane dynamics over length (~100 nm) and time (~1–1,000 ns) scales corresponding to key membrane processes, including membrane trafficking, viral budding, tubulation, and vesiculation (Reynwar et al., 2007; Tian et al., 2009; Bradley and Radhakrishnan, 2016). These length and time scales also correspond with the conformational dynamics of membrane proteins (Perozo et al., 2002; Henzler-Wildman and Kern, 2007), making NSE well-suited for studies of protein/membrane interactions and understanding the role of membrane dynamics in regulating protein function and spatiotemporal signaling. Indeed, NSE studies on lipid membranes over the last few years have provided remarkable insights into the fundamental biophysical laws that govern nanoscopic membrane dynamics as well as, the interaction of membranes with inclusions and with their surrounding environment.

To date, the majority of NSE studies target membrane bending properties using protiated ULVs in D_2_O, thus taking advantage of the amplified contrast between the membrane and its deuterated surrounding. NSE detects these fluctuations in the form of decays in the intermediate dynamic structure factor, *I*(*q, t*)/*I*(*q*, 0), as a function of the Fourier time, *t* (Figure 8B). The obtained signals are generally interpreted using the elastic sheet model with a stretched exponential function (Helfrich, 1973): $I\left(q,t\right)/I\left(q,0\right)=\exp-{\left(\Gamma \left(q\right)t\right)}^{\frac{2}{3}}$, yielding the q-dependent decay rates, Γ(*q*), of the measured fluctuations. In NSE measurements of bending fluctuations, Γ(*q*) typically exhibits a *q*^3^ dependence (Figure 8C), as predicted by Zilman and Granek for thermally undulating elastic thin sheets, such that (Zilman and Granek, 1996):

(1) $$\begin{array}{l} \Gamma {\left(q\right)}_{bend}\left(q\right)=0.025\alpha \frac{k_{B}T}{\eta_{sol}}\sqrt{\frac{k_{B}T}{\tilde{\kappa }}}q^{3}, \end{array}$$

where *k*_*B*_*T* is the thermal energy, η_*sol*_ is the solvent viscosity, and α is a parameter that results from angular averaging of $\vec{q}$ relative to the membrane normal. Such theoretical analysis of NSE data enables direct calculations of an important membrane property, i.e., the effective bending rigidity modulus, $\tilde{\kappa }$. With these powerful capabilities in mind, NSE has become an important neutron scattering tool to answer questions related to membrane dynamics.

In recent years, NSE has been used to a great effect to measure the bending moduli of lipid membranes with various degrees of complexity, ranging from single component to multi-component lipid membranes that more closely resemble biological cell membranes. For instance, bending rigidity measurements of membranes comprised of lipids with different acyl chain lengths and unsaturations (Yi et al., 2009; Brüning et al., 2014; Nagao et al., 2017) have provided important insights into how the physicochemical properties of the membrane affect its mechanical properties when paired with structural information, such as membrane thickness or molecular packing, as discussed earlier (Kučerka et al., 2009b). Collectively, these measurements can shed new light on membrane structure-property relations and how they influence membrane function. This topic was recently explored in two NSE studies on cholesterol-rich lipid membranes by Chakraborty et al. (2020) and on binary lipid membranes with hydrophobic lipid mismatch by Kelley et al. (2020) Both studies found strong evidence that membrane mechanics scale with the AL, emphasizing the strong interdependence of the structural and dynamical properties in lipid membranes.

Notably, using NSE Chakraborty et al. (2020) resolved a long debated question about the role of cholesterol in membrane mechanics, one that has far-reaching implications with regard to membrane function. They discovered that cholesterol has a non-trivial local stiffening effect on membranes with unsaturated lipids commonly found in cell membranes (Chakraborty et al., 2020). Their results showed that on the collective molecular level (i.e., small molecular assemblies), membrane mechanics scale with cholesterol-induced “densification” of the membrane, as can be deduced from membrane structure-property relations. However, these conclusions contradicted previous studies reporting null mechanical effects of cholesterol in unsaturated lipid membranes when measured over extended spatial and temporal scales (Pan et al., 2009; Gracia et al., 2010), suggesting the presence of scale-dependent membrane dynamics. This notion of dynamical hierarchy in membranes is not surprising or new, but is experimentally challenging due to the difficulty in reconciling dynamics over very different length scales (ranging from molecular to macroscopic length scales). NSE thus offers great potential in bridging these two length scales through direct experimental observations of membrane dynamics that are intermediate between molecular motions and continuum deformations.

Other applications of NSE spectroscopy include studies involving changes in membrane bending rigidity in response to additive molecules, such as trehalose (Brüning et al., 2014), melittin (Sharma et al., 2015; Sharma and Qian, 2019), and inorganic nanoparticles used in drug delivery (Chakraborty et al., 2018). These studies provide a nanoscale perspective of how small molecules could affect the softening or stiffening of lipid membranes under biologically relevant conditions and in targeted applications. NSE has also been used to interrogate the mechanical response of membranes to environmental conditions, including temperature (Brüning et al., 2014), pH (Boggara et al., 2010), and the presence of surrounding macromolecules (Yu et al., 2020). As a result, this body of experiments is now starting to shape our understanding of how membranes respond to external cues that mimic the different conditions experienced by cell membranes, a current area of research.

**Figure 9:**
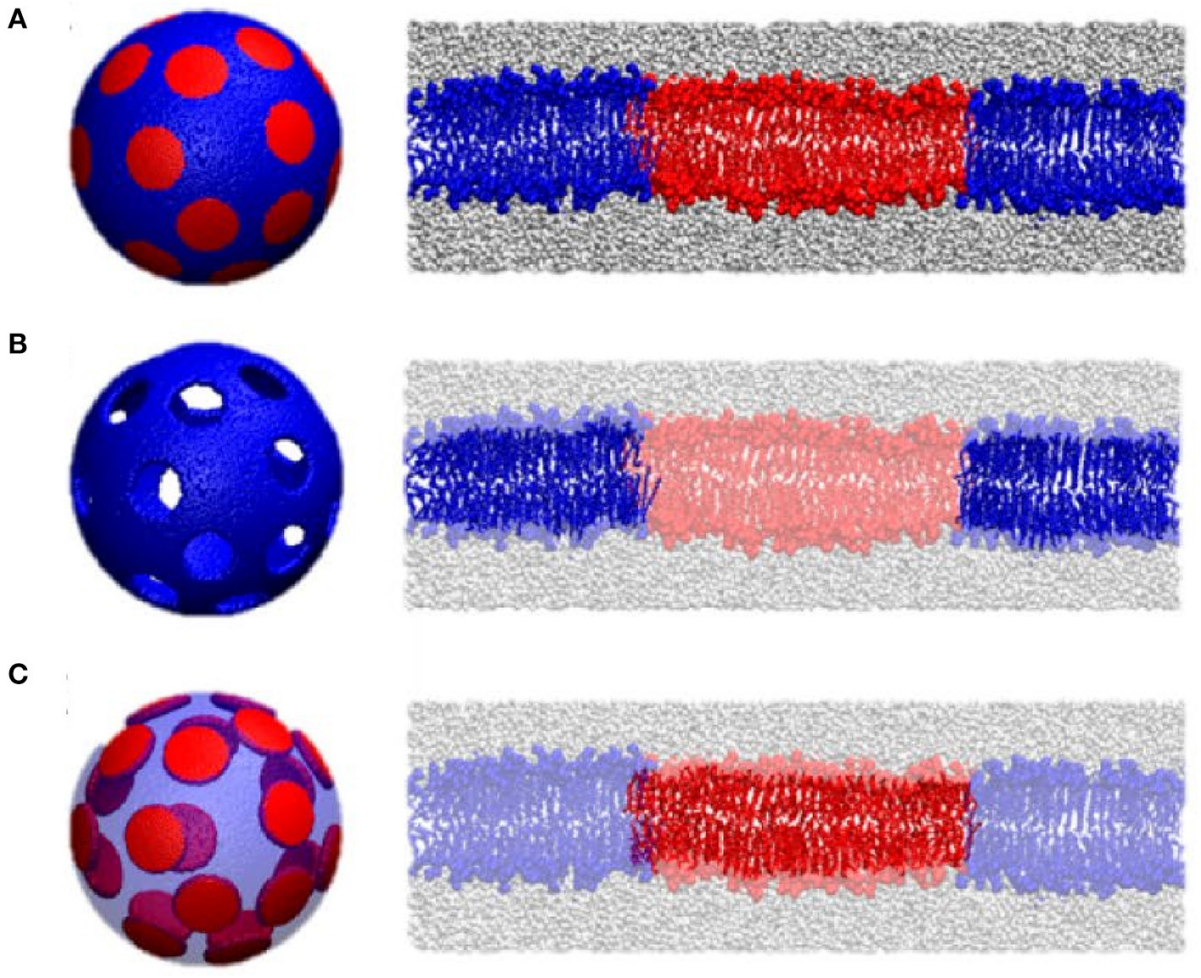
Contrast matching used to study the mechanical properties of lipid domains in 60 nm diameter ULV model membranes. Three contrast matching schemes were used to change the neutrons sensitivity of lipid domains in model membranes. They were: **(A)** no contrast matching, where the entire ULV is visible to neutrons; **(B)** domains contrast matched to the solvent in order to highlight the surrounding membrane; and **(C)** the membrane is contrast matched to the solvent highlighting the nanodomains; Transparent colors indicate contrast matching to the solvent using these three different contrast matching schemes. Figure adapted from Nickels et al. (2015).

Other applications of NSE involve the utilization of neutron isotope sensitivity to investigate the dynamics of selective features within lipid membranes. For example, Rickeard et al. (2020) used asymmetric lipid membranes with one of their bilayer leaflets selectively deuterated to investigate the effect of asymmetry and leaflet coupling on membrane bending fluctuations. Surprisingly, they found that asymmetric membranes possessed a larger bending modulus than symmetric bilayers with the same average lipid composition, a result that was uniquely enabled by NSE. In an earlier study, Nickels et al. (2015) used a combination of protiated and deuterated lipids to generate lateral contrast within domain-forming lipid membranes in order to study the mechanical properties of the nanoscopic lipid domains populating 60-nm diameter ULVs (Figure 9). By adjusting the percent D_2_O, they were able to perform NSE measurements targeting the nanoscopic lipid heterogeneities in ULVs in order to study their bending rigidity.

Isotope labeling has also been used in NSE experiments to access thickness fluctuations in lipid membranes by using tail-deuterated membranes, such that the acyl chain region of the membrane matches the SLD of the solvent or buffer. This fluctuation mode has long been predicted but was only recently observed experimentally with NSE (Nagao, 2009; Woodka et al., 2012), where thickness fluctuations manifest themselves as enhanced dynamics in addition to the bending fluctuations (Figure 8D). The dynamics are most pronounced at *q*-values that correspond to the membrane thickness, as was recently verified by coarse-grained MD simulations (Carrillo et al., 2017). Analysis of this dynamic mode using an approach developed by Nagao et al. (2017) and based on the theoretical framework of membrane fluctuations by Bingham et al. (2015) has enabled the extraction of other biophysical membrane parameters namely, membrane viscosity. With these recent advances, NSE is rapidly becoming the tool of choice when it comes to understanding membrane dynamics and gaining new biophysical insights.

## 4. Concluding Remarks

Although much research has been focused at addressing the structure–function relationship of biological membranes, there are many open questions that remain. Nevertheless, what has become clear over the last 10 years, or so, is that neutron scattering techniques are invaluable tools in studies of biological or biologically relevant membranes. Neutron scattering techniques offer a range of capabilities, some of which are unique to them. For instance, the ability to easily alter the neutron contrast of the sample enabled Heberle et. al. to study nanoscopic lipid domains and relate thickness mismatch between the domains and their surrounding to line tension theories (Heberle et al., 2013). This approach by Heberle et al. was then extended by Nickels et al. to separately study the bending rigidities of the lipid domains populating the membrane and their surrounding lipids using NSE (Nickels et al., 2015).

The ability to reconcile different contrast x-ray and neutron scattering data using a single model was addressed by the CSM approach of Wiener and White (1991b). However, it took almost 20 years to develop an approach whereby, multiple sets of different contrast data were fitted using a robust model guided by MD simulations (Kučerka et al., 2008). Importantly, the approach by Kučerka et al. made use of fully hydrated ULVs, model systems that are considered excellent mimics of the PM.

Neutron spin echo, a technique capable of interrogating membrane dynamics over spatial and temporal scales relevant to critical biological functions is rapidly becoming the technique of choice when it comes to measuring the mechanical properties of membranes. Combined with different deuteration strategies, NSE enables non-invasive measurements of collective membrane dynamics that would otherwise be challenging to explore using traditional spectroscopic methods. When combined with MD simulations, which are sensitive to similar length and time scales as NSE, a molecular level understanding of the different components making up membranes can be achieved (Nickels et al., 2015; Chakraborty et al., 2020). Moreover, NSE studies combined with SDP analysis can be used to further refine the currently used MD force fields and aid in the development of new experimental and computational approaches (Doktorova et al., 2019b).

In summary, we envision the different neutron scattering techniques playing leading roles in understanding the static and dynamic structures of membranes. When combined with MD simulations, neutron scattering techniques offer the possibility of providing unique insights into individual molecules making up membranes and micrometer patches of membrane comprised of thousands of individual molecules. Complementary dynamic studies will range from picoseconds to tens of microseconds. In particular, NSE data analysis is still in its infancy, and developing new physical models to interpret the data will enable NSE to become a mainstream technique. Finally, in order to make neutron scattering more relevant to biologists, it needs to expand, with greater frequency, into the study of fully functioning biological systems, similar to the one reported by Nickels et al. (2017).

## Author Contributions

JJK, HLS, RA, and JK wrote the manuscript. All authors contributed to the article and approved the submitted version.

## Conflict of Interest

The authors declare that the research was conducted in the absence of any commercial or financial relationships that could be construed as a potential conflict of interest. The reviewer NK declared a past co-authorship with the authors JJK, HS, JK to the handling Editor.
